# Clinical Relevance of Pathogens Detected by Multiplex PCR in Blood of Very-Low-Birth Weight Infants with Suspected Sepsis – Multicentre Study of the German Neonatal Network

**DOI:** 10.1371/journal.pone.0159821

**Published:** 2016-07-29

**Authors:** Birte Tröger, Christoph Härtel, Jan Buer, Michael Dördelmann, Ursula Felderhoff-Müser, Thomas Höhn, Nico Hepping, Georg Hillebrand, Angela Kribs, Janina Marissen, Dirk Olbertz, Peter-Michael Rath, Susanne Schmidtke, Jens Siegel, Egbert Herting, Wolfgang Göpel, Joerg Steinmann, Anja Stein

**Affiliations:** 1 Department of Pediatrics at the University of Lübeck, Lübeck, Germany; 2 Institute of Medical Microbiology, University of Duisburg-Essen, Essen, Germany; 3 Department of Pediatrics at Diakonissen Hospital Flensburg, Flensburg, Germany; 4 Department of Pediatrics, University Hospital Essen, University Duisburg-Essen, Essen, Germany; 5 Department of Pediatrics at the University of Düsseldorf, Düsseldorf, Germany; 6 Department of Pediatrics at St. Marien Hospital Bonn, Bonn, Germany; 7 Department of Pediatrics at Hospital of Itzehoe, Itzehoe, Germany; 8 Department of Pediatrics at the University of Cologne, Cologne, Germany; 9 Neonatology Unit at Südstadt Hospital Rostock, Rostock, Germany; 10 Neonatology Unit at Asclepios Hospital Hamburg-Barmbek, Hamburg, Germany; 11 Neonatology Unit at the Children´s Hospital Hannover Auf der Bult, Hannover, Germany; The University of Hong Kong, HONG KONG

## Abstract

**Introduction:**

In the German Neonatal Network (GNN) 10% of very-low-birth weight infants (VLBWI) suffer from blood-culture confirmed sepsis, while 30% of VLBWI develop clinical sepsis. Diagnosis of sepsis is a difficult task leading to potential over-treatment with antibiotics. This study aims to investigate whether the results of blood multiplex-PCR (SeptiFast^®^) for common sepsis pathogens are relevant for clinical decision making when sepsis is suspected in VLBWI.

**Methods:**

We performed a prospective, multi-centre study within the GNN including 133 VLBWI with 214 episodes of suspected late onset sepsis (LOS). In patients with suspected sepsis a multiplex-PCR (LightCycler SeptiFast MGRADE-test^®^) was performed from 100 μl EDTA blood in addition to center-specific laboratory biomarkers. The attending neonatologist documented whether the PCR-result, which was available after 24 to 48 hrs, had an impact on the choice of antibiotic drugs and duration of therapy.

**Results:**

PCR was positive in 110/214 episodes (51%) and blood culture (BC) was positive in 55 episodes (26%). Both methods yielded predominantly *coagulase-negative staphylococci (CoNS)* followed by *Escherichia coli* and *Staphylococcus aureus*. In 214 BC—PCR paired samples concordant results were documented in 126 episodes (59%; n = 32 were concordant pathogen positive results, n = 94 were negative in both methods). In 65 episodes (30%) we found positive PCR results but negative BCs, with CoNS being identified in 43 (66%) of these samples. Multiplex-PCR results influenced clinical decision making in 30% of episodes, specifically in 18% for the choice of antimicrobial therapy and in 22% for the duration of antimicrobial therapy.

**Conclusions:**

Multiplex-PCR results had a moderate impact on clinical management in about one third of LOS-episodes. The main advantage of multiplex-PCR was the rapid detection of pathogens from micro-volume blood samples. In VLBWI limitations include risk of contamination, lack of resistance testing and high costs. The high rate of positive PCR results in episodes of negative BC might lead to overtreatment of infants which is associated with risk of mortality, antibiotic resistance, fungal sepsis and NEC.

## Introduction

Sepsis continues to be a major cause of mortality and long-term morbidity in very-low-birth-weight infants (VLBWI, birth weight < 1500 g). Under well-standardized neonatal intensive care unit conditions the incidence of blood culture (BC) proven sepsis in VLBWI is 10–20% while 30–50% of VLBWI develop clinical sepsis [1, 2]. Early diagnosis of sepsis and prompt empirical therapy with anti-infective drugs are often life-saving. Since highly reliable biomarkers for sepsis in VLBWI are lacking, antibiotic treatment is often initiated because of unspecific clinical signs alone. If the treatment is continued for at least 5 days without BC-confirmed pathogenic growth, diagnosis of “clinical sepsis” is made [3, 4]. BC remains the `gold-standard`for the diagnosis of bloodstream infection. Its sensitivity, however, is limited in VLBWI due to small sample volume, pre-treatment with antibiotics or intermittent bacteremia. In addition to that, results of BCs may take up to 48–72 hours until positive results can be reported [5, 6]. Advances in molecular microbiology have provided culture-independent molecular assays for rapid diagnosis of the causative agent of infection. PCR-assays developed for specific detection of pathogens in the blood were described as early as 1993 [7]. The development of broad-spectrum PCR-assays facilitates more universal detection of microorganisms [8]. In adult patients with suspected sepsis rapid detection of the 25 most common sepsis pathogens (bacterial and fungal) in blood by nucleic acid amplification (e.g. SeptiFast^®^) has had relevant impact on subsequent management [9, 10]. In neonates detection of bacterial DNA in blood samples is suggested to represent a rapid method in diagnosing sepsis [11, 12]. In line with that, Kasper et *al*. demonstrated that PCR results are reliable from micro volume samples as small as 100 μl [13]. However, it is largely unknown to what extent availability of PCR results would influence clinical decision making. In the present multicentre study, we evaluated the clinical value of a multiplex-real-time-PCR assay in episodes of suspected late-onset sepsis in VLBWI.

## Methods

### Study population

The German Neonatal Network (GNN) is a well-established, prospective multicentre collaboration. This prospective study was offered to all study centres during the two year study period (1^st^ of May 2012 until 30^th^ of April 2014). Eleven centres participated and sent samples of VLBWI with clinically suspected sepsis ≥ 72 hours of life after informed written parental consent. Samples were analysed at the Institute of Medical Microbiology, University of Duisburg-Essen. A predefined clinical and laboratory data set of 62 parameters was recorded for each patient on clinical record files (onset of symptoms, clinical and laboratory signs of infection, devices (central or peripheral venous lines, endotracheal tubes, non-invasive ventilation), nutrition, pathogens, site of pathogen detection, diagnosis, antimicrobial therapy, therapy adjustment and other treatments). Data quality was approved by regular on-site monitoring of participating centres by a physician trained in neonatology including cross-check of local microbiological read-outs. In the tables, parameters were included with > 90% full clinical data sets available.

### Inclusion criteria

GNN-VLBWI (birth weight < 1500 g and gestational age ≤ 36+6 weeks) with an episode of suspected sepsis ≥ 72 hours of life (LOS, late onset sepsis) were included when blood culture results and multiplex-PCR results were available. Infants with lethal abnormalities and exclusively early onset sepsis (EOS, sepsis within the first 72 hours of life) were not considered for this study.

### Definitions and main outcome

For the diagnosis "sepsis" the definitions of the Surveillance Protocol NEO-KISS of Robert-Koch Institute (RKI) were used in preterm infants (www.nrz-hygiene.de) Specifically, BC confirmed sepsis was defined as clinical sepsis with proof of causative agent in BC. If coagulase-negative staphylococci (CoNS) were isolated as single pathogen in one peripheral BC, two clinical signs and one laboratory sign (platelet count < 100/nl, C-reactive protein > 20 mg/L, immature/total neutrophil ratio > 0.2, white blood cell count < 5/nl) were required for classification of *CoNS* sepsis. Diagnosis of "clinical sepsis" without detection of pathogens in BC was based on 2 clinical parameters, no other focus and the need for at least 5 days of anti-infective treatment [3, 4]. In patients with suspected sepsis a multiplex-PCR (LightCycler SeptiFast MGRADE-test^®^) was performed from 100 μl EDTA blood in addition to center-specific laboratory biomarkers. The attending neonatologist documented whether the PCR-result, which was available after 24 to 48 hrs during weekdays, had an impact on the choice of antibiotic drugs and duration of therapy (main outcome).

### Sample collection and transport

In patients with suspected LOS centre-specific laboratory parameters (such as interleukin 6, C-reactive protein, full blood count) and a BC were taken. In addition, a blood sample for the multiplex-PCR (SeptiFast-test^®^) was obtained (100–200 μl EDTA blood was collected in DNA-free Sarstedt Microvette^®^ tubes, Nümbrecht, Germany). Blood samples were taken by peripheral venipuncture. To ensure antiseptic blood collection conditions, skin disinfection with octenidine 0.1% with an exposure time of 60 sec was recommended in VLBWI. The BC was sent as usual to the attending microbiological laboratory. Blood samples for multiplex-PCR were immediately stored at 3–7 degrees Celsius and sent with cooling elements to the Institute of Medical Microbiology, University of Duisburg-Essen, Essen, Germany. Transportation time varied between 6–24 hours.

### Multiplex-PCR

The SeptiFast^®^-test (LightCycler^®^ Roche Diagnostics, Mannheim, Germany) uses real-time multiplex PCR to detect more than 20 different pathogens in blood [14]. The diagnostic probes for PCR target the internal transcribed sequences situated between 16S and 23S bacterial ribosomal RNA as well as between 18S and 5.6S fungal ribosomal RNA [15]. The limits for detection of skin bacteria as CoNS and *Streptococcus* spp. were 100 CFU/ml and 30 CFU/ml for all other pathogens. The protocol has been adapted especially for small amounts of blood from 100 μl and contains control samples, which can exclude contamination during preparation. To ensure quality of methodology, tests regarding DNA contamination were performed using normal saline. Multiplex-PCR detected no DNA.

### Ethical approval

Ethical approval was given by the University of Lübeck Ethical Committee and local ethical committees at the other study centers. Specifically: Ethical Board of the University of Kiel, Ethical Board of the University of Essen, Ethical Board of the University of Düsseldorf, Ethical Board of the University of Bonn, Ethical Board of the Medical Chamber of Hamburg, Ethical Board of the University of Cologne, Ethical Board of the Medical Board of the University of Rostock, Ethical Board of the Medical School Hannover. Informed written consent was given by parents (as legal representatives) on behalf of their infants.

### Statistical analysis

Data analysis was performed using the SPSS 20.0 data analysis package (Munich, Germany). The chi-square test, Fisher´s exact test and Mann–Whitney U test were applied for statistical analysis of differences between different groups. The level of significance was defined as p<0.05 in single comparisons.

## Results

### Clinical characteristics and signs of infection

In Table 1, the clinical characteristics of the multicentre study cohort (n = 133 VLBWI, 214 LOS episodes) are described.

**Table 1:** Clinical characteristics of the VLBW cohort with suspected LOS.

	VLBWI with suspected LOS
**Number of infants**	133
**Number of LOS—episodes**	214
**Gestational age** (median/interquartile range in weeks)	25.6 / 24.5–28.4
**Birth weight** (median/interquartile range in grams)	730 / 555–1030
**Gender** (male, %)	46.5
**Onset of clinical symptoms** (median/interquartile range in days)	19 / 10–41
**Clinical signs of infection (%)**	
Desaturations	63.3
Increased numbers of bradycardias < 80 bpm	53.5
Increased numbers of apneas > 20 sec	38.6
Grey skin colour	33.5
Abdominal distension	33.5
Apathy/lethargy	33.0
Temperature instability	28.4
Fever	28.4
Capillary refill time > 2 sec	27.0
Tachypnea	27.0
Tachycardia > 200 bpm	20.9
Reduced feeding tolerance	12.1
Vomiting	10.7

Median gestational age of VLBWI was 25.6 weeks and median birth weight was 730 grams. Onset of infection symptoms occurred at a median of 19 days of life. The predominant clinical symptoms for LOS were increased number of desaturations (63.3%) and increased numbers of bradycardias (53.5%), respectively. These clinical signs were accompanied by blood glucose level > 140 mg/dl (25.6%) and elevated C-reactive protein levels (43.7%; Table 2). C-reactive protein levels were documented as maximum levels during LOS episodes and ranged from 3 mg/l to 280 mg/l.

**Table 2:** Laboratory signs of infection of the VLBW cohort with suspected Late-onset sepsis (LOS).

Laboratory signs of infection (%)	VLBWI with suspected LOS
C-reactive protein > 20 mg/l	43.7
Blood glucose level > 140 mg/dl	25.6
Interleukin-6 elevated	18.6
Thrombocytopenia < 100/nl	15.3
I/T-Ratio > 0.2	14.4
Base excess < -10 mval/l	8.8
Leukopenia <5/nl	6.5

Based on NEOKISS definitions, sepsis episodes were finally classified as clinical sepsis (27.9%), *CoNS* sepsis (13.0%) and BC proven sepsis with other bacteria (9.8%) (Table 3).

**Table 3:** Final diagnosis of the VLBW cohort with suspected LOS episodes.

Final diagnosis	VLBWI with suspected LOS (%)
Clinical sepsis	27.9
Coagulase negative Staphylococci (CoNS)-sepsis	13.0
Blood culture positive sepsis	9.8
Focal intestinal perforation (FIP)	4.2
Necrotizing enterocolitis (NEC)	3.7
Histologically confirmed FIP/NEC	5.1
Pneumonia	5.6
Meningitis	0.9
Other infectious focus	1.4
No infection	33.5

### Isolated pathogens

Multiplex-PCR was positive in 110 episodes (51%) and BC was positive in 55 episodes (26%) (Table 4).

**Table 4:** Pathogens identified by multiplex-PCR or blood culture.

Species	Multiplex-PCR (n/%)	Blood culture (n/%)
Coagulase-negative Staphylococci (CoNS)	73 / 57.4	36 / 65.4
*Escherichia coli*	11 / 8.6	7 / 12.7
*Staphylococcus aureus*	11 / 8.6	6 / 10.9
*Enterobacter cloacae*	10 / 7.8	1 / 1.8
*Enterococcus faecalis*	7 / 5.5	3 / 5.4
*Klebsiella pneumoniae*	7 / 5.5	0
*Streptococcus* spp.	5 / 3.9	0
*Aspergillus fumigatus*	1 / 0.7	0
Corynebacteriae*	0	1 / 1.8
Bifidobacteriae*	0	1 / 1.8
**Total**	125#	55

^#^15 multiplex-PCR samples yielded polymicrobial results which are included in the table

*Species which cannot be detected by multiplex PCR

15 samples showed polymicrobial positive multiplex-PCR. In 7 out of 15 multiplex-PCR samples BC detected no pathogen, in 7 out of 15 samples one of the pathogens detected with multiplex-PCR was found in BCs as well. In the remaining sample BC and multiplex-PCR revealed different pathogens. The pathogenic spectrum is outlined in Table 4. Both methods yielded predominantly CoNS followed by *Escherichia coli* and *Staphylococcus aureus*. BCs revealed more often *Corynebacteriae* and *Bifidobacteriae*.

### Concordance of multiplex-PCR and blood culture results

In 214 BC—PCR paired samples concordant results were documented in 126 episodes (n = 32 were concordant pathogen positive results, n = 94 were negative in both methods). In 65 episodes (30.3%) we found positive PCR results but negative BCs, with CoNS being identified in 43 (66.1%) of these samples (Table 5). In those episodes the clinicians made the following final diagnoses: no infection in 23 episodes, clinical sepsis in 22 episodes, CoNS sepsis in 9 episodes and other entities in 11 episodes. Detailed information is given in Table 6. In 13 episodes multiplex-PCR and BC were positive, but the results were not concordant. In 10 samples with positive BC, multiplex-PCR yielded negative results. Pathogens detected were CoNS (n = 9) and *Enterococcus faecalis* (n = 1). Only one case with CoNS-positive BC and negative multiplex-PCR was classified as `no infection`and CoNS was considered as contaminant. All other BC results represented clinically relevant infections. A fungal pathogen was detected only once with multiplex-PCR and was considered as contamination.

**Table 5:** Pathogens identified by multiplex-PCR with negative blood culture result.

Species	Multiplex-PCR (n)
Coagulase-negative Staphylococci (CoNS)	43
*Staphylococcus aureus*	4
*Enterobacter cloacae*	6
*Enterococcus faecalis*	4
*Klebsiella pneumoniae*	3
*Streptococcus* spp.	4
*Aspergillus fumigatus*	1
Total	65 #

^#^5 multiplex-PCR samples yielded polymicrobial results which are included in the table

**Table 6:** Pathogens identified by multiplex-PCR with negative blood culture result and corresponding final diagnosis.

Species	No infection (n)	Clinical sepsis (n)	CoNS sepsis (n)	Other entities (n)
Coagulase-negative Staphylococci (CoNS)	17	9	9	8 (n = 2 pneumonia; n = 2 abdominal wound infection; n = 3 unspecific viral infection; n = 1 CNS infection
*Staphylococcus aureus*	1	2		1 (n = 1 pneumonia)
*Enterobacter cloacae*	3	3		
*Enterococcus faecalis*		2		2 (n = 2 unspecific viral infection)
*Klebsiella pneumoniae*		3		
*Streptococcus* spp.	1	3		
*Aspergillus fumigatus*	1			
Total (n)	23	22	9	11

### Treatment adjustments

In 208/214 episodes clinicians had documented whether multiplex-PCR had an impact on the choice of antibiotic drugs and/or duration of therapy. As such, multiplex-PCR results influenced clinical decision making in 63 out of 208 episodes with suspected LOS (30%). Choice of antimicrobial therapy was influenced in 37 of 208 (18%) and duration of antimicrobial therapy in 46 of 208 LOS episodes (22%). In episodes with both BC and multiplex-PCR concordant positive result (n = 30 with available information on treatment adjustments), multiplex-PCR influenced clinical decision making in 12 episodes (40%), specifically choice of antimicrobial therapy in 10 episodes (30%) and duration of antimicrobial therapy in 5 out of 30 episodes (17%). The influence of multiplex-PCR result was mainly due to the earlier result compared to BC. Regarding episodes with positive multiplex-PCR and negative BC (n = 62 (n = 62 with available information on treatment adjustments), multiplex-PCR results were important for therapy in 27 out of 62 episodes (42%), for choice of antimicrobial therapy in 18 out of 62 episodes (29%) and in 16 out of 62 (25%) for the duration of therapy.

## Discussion

Appropriate and timely antibiotic treatment in LOS is crucial to reduce morbidity and mortality in VLBWI. Here, we present a multi-centre study investigating the clinical relevance of multiplex-PCR (SeptiFast^®^-test) results in VLBWI with episodes of suspected LOS sepsis. Our data imply that multiplex-PCR is feasible from low-volume samples of VLBWI with suspected LOS in a multi-center setting. Moreover, integration of multiplex-PCR data into clinical routine has a moderate impact on therapeutic judgement. BCs were positive in 26% of all samples in comparison to 51% with multiplex-PCR. The higher rates of pathogen detection using multiplex-PCR in comparison to BCs have been described in various studies [11, 12, 16]. BC positivity data were compatible with previous studies in VLBWI cohorts [2, 17–19]. Concordance between BC and multiplex-PCR (59%) results proved to be lower than in other studies, which reported concordance > 75% [9, 20]. Multiplex-PCR was negative in 10 episodes with positive blood culture. Only one case with CoNS-positive BC and negative multiplex-PCR was classified as `no infection`and CoNS was considered as a contaminant. All other BC results represented clinically significant infections. It is important to note, that all pathogens identified in BCs are part of the multiplex-PCR spectrum. This may possibly have occurred due to genetic variability among the pathogens, or mutations of the target site, inappropriate sample preparation or prolonged transport times. We evaluated if transportation time could have had an influence on multiplex-PCR detection rate. Four samples with positive BC and negative multiplex-PCR were sent from the Department of Pediatrics, University Hospital of Essen and multiplex-PCR was performed at the Institute of Medical Microbiology, University of Essen which is located at the same campus. Transportation time of these samples was the shortest, making an influence of transportation time unlikely. Other samples with positive BC and negative multiplex-PCR were sent from Düsseldorf (n = 3), Cologne (n = 1) and Lübeck (n = 2). Transportation time was between 6–24 hours and did not differ with respect to the other centers. The “sensitivity” aspect may also be related to PCR detection limits of skin bacteria. CoNS and *Streptococcus* spp. were detected only at 100 CFU/ml and the sensitivity limit was 30 CFU/ml for all other pathogens (set up by the manufacturer to avoid detection of contaminants). In 30.3% of LOS-episodes we found positive multiplex-PCR results and corresponding negative BCs. It has been described that the use of molecular assays has the risk of detecting contaminant DNA, thereby producing false-positive results [8]. Despite several advantages of PCR-based methods, a potential limitation of these molecular assays is the detection of DNA from dead/non-viable microorganisms, resulting in false-positive results and potentially leading to overuse of antibiotics which is associated with the risk for antibiotic resistance, fungal sepsis, NEC and mortality [21, 22, 23]. This is a clinically relevant issue, although a recent report on multiplex-PCR detection of pathogen DNA in blood has convincingly related DNA presence to the actual infection status of the patient [24]. Hence interpretation of multiplex-PCR results should always be assessed in a context with other laboratory data and clinical conditions of the patient. In our study CoNS was the main pathogen identified in 66% of PCR-positive/BC-negative samples. In 35% of cases with positive multiplex-PCR and negative BCs clinicians finally decided that no infection was present. In 34% diagnosis of clinical sepsis and in 14% diagnosis of CoNS-sepsis was made. Different study groups observed higher rate of positive results by multiplex-PCR compared to BC in patients undergoing antimicrobial therapy [11]. Tschiedel et *al*. [25] showed in 2012 that detection rate via blood culture in paediatric patients under antibiotic therapy was only half as high as in patients without antimicrobial treatment. It is a known problem, that cultivation of bacteria and fungi under anti-infective therapy is rarely successful [16]. In our study, there was no exact evaluation regarding prior therapies and residuals of antimicrobials in blood samples. Importantly, multiplex-PCR data were available 24-48h after blood withdrawal during week-days. The results led to adjustment of initial therapy in 30% of VLBWI with suspected LOS. Therapy modifications were more often performed when *CoNS* were detected by multiplex-PCR. However, whether therapeutic adjustments initiated by the multiplex-PCR had an impact on clinical outcomes was not determined in this study. Our study has some limitations. It was a non-randomized assessment, and therefore a selection bias cannot be excluded which may have influenced the rate and spectrum of pathogens found in our cohort. We did not have an exact evaluation regarding antimicrobial pre-treatment, although VLBWI are often managed with empirical antibiotic therapy and results of BCs may be influenced by antimicrobial therapy. Evaluation of multiplex-PCR in pre-treated VLBWI with suspected LOS including assessment of antimicrobial remnants (in blood or urine) would be an important future project. Patient’s characteristics in our study are comparable to those of other sepsis studies in VLBWI [2] and pathogens identified were characteristic for the group of VLBWI and their risk factors. Another limitation results from the fact that sample volumes for BC were not standardized (0.1-1ml). It is known that the sensitivity of BC results correlates with the acquired blood volumes and the number of inoculated bottles. Costs for multiplex-PCR testing are approximately 5 times as high as for a pair of BCs and specially trained staff is necessary to perform multiplex-PCR. The most important advantage of BC over multiplex-PCR is, that susceptibility testing of an isolate can be performed, allowing the implementation of specifically targeted antimicrobial or antifungal therapy. BC also has an advantage over multiplex-PCR with respect to microorganisms not included. However, bacteria or fungi which were not included in the SeptiFast pattern have only been detected twice.

## Conclusion

Addition of multiplex-PCR to conventional BCs had a moderate impact on clinical management in about one third of LOS-episodes. The main advantage of multiplex-PCR was the rapid detection of pathogens from micro-volume blood samples. In preterm infants limitations include limited spectrum of pathogens, risk of contamination, lack of resistance testing and high costs. Specifically, the high rate of positive PCR results in episodes of negative blood culture might lead to overtreatment of infants which is associated with risk of mortality, antibiotic resistance, fungal sepsis and NEC.
